# Implementation of a Point-of-Care Blood Testing System in a Canadian Emergency Department: A Mixed-Methods Evaluation of Workflow and Clinician Perceptions

**DOI:** 10.7759/cureus.114077

**Published:** 2026-08-06

**Authors:** Sarah Allam, Melissa McGowan, Brodie Nolan

**Affiliations:** 1 Faculty of Medicine, University of Toronto, Toronto, CAN; 2 Department of Emergency Medicine, St. Michael's Hospital, Toronto, CAN; 3 Division of Emergency Medicine, Faculty of Medicine, University of Toronto, Toronto, CAN

**Keywords:** blood gas analysis, clinician-reported outcomes, emergency department workflow, point-of-care testing, turnaround time

## Abstract

Objective: Point-of-care blood gas and chemistry testing is commonly used in emergency care to provide rapid biochemical information during initial patient assessment. We evaluated the implementation of the Siemens Healthineers ePOC Blood Analysis System in a tertiary-care Canadian emergency department (ED) by assessing clinician-perceived usability, adequacy of training, satisfaction, and perceived effects on workflow and clinical decision-making.

Methods: This quality-improvement and implementation evaluation was reported in accordance with the Standards for Quality Improvement Reporting Excellence (SQUIRE) 2.0 guidelines, where applicable. The evaluation consisted of (1) a retrospective analysis of core laboratory turnaround times (TATs) during the early implementation period and (2) a post-implementation survey assessing clinician-reported usability, adequacy of training, satisfaction, and perceived effects on workflow efficiency and clinical decision-making.

Results: From March to April 2025, the device was used in 42 adult ED patients; 28/42 (66.7%) cases had core laboratory TAT data available. The mean core laboratory TAT was 18 minutes (range: 0-33) for blood gas syringe samples and nine minutes (range: 0-19) for plasma samples. From August to October 2025, 32 clinicians completed the survey. Most respondents reported using ePOC at least monthly (31/32, 96.9%), and 21/32 (65.6%) rated it as easy or very easy to use. Among 29 respondents to the training item, 16 (55.2%) felt training was adequate, while eight (27.6%) felt it was inadequate. Reported benefits included improved efficiency of blood analysis (24/30, 80.0%), shorter time to critical results (25/31, 80.6%), and faster clinical decision-making (25/30, 83.3%). Overall, 24/32 (75.0%) respondents were satisfied or very satisfied, and 29/32 (90.6%) supported continued ED use. Reported barriers included calibration delays, lack of direct electronic medical record integration, and perceived concerns regarding agreement with laboratory values.

Conclusion: Clinician responses were generally supportive of ePOC use, but perceptions of inadequate training and workflow challenges suggest that its benefits may depend on a more structured implementation approach.

## Introduction

Bedside blood gas point-of-care testing (POCT) rapidly measures selected blood gas, electrolyte, and metabolite parameters [1]. Bypassing the delays associated with centralized laboratory processing, POCT in the emergency department (ED) can support early risk stratification and clinical decision-making [2-5]. In a 2024 Spanish multicenter randomized clinical trial, POCT implementation was associated with shorter laboratory turnaround time (TAT), shorter time to clinical decision, and shorter ED length of stay [3].

However, observational studies have suggested that effective implementation of POCT in practice remains variable. A 2025 cross-sectional survey of all 71 Swedish EDs found universal adoption of POCT, yet guidelines were present in only 44% of sites, and staff training practices varied considerably [6]. The use of POCT has been noted to rely on operational factors, including perceived accuracy, ease of use, and accessibility [7]. Similarly, Canadian guidance has maintained that POCT deployment in a clinical setting is best when supported by a robust quality assurance framework with defined clinical needs [8,9].

To evaluate local implementation, we assessed clinician-perceived usability, adequacy of training, satisfaction, and perceived effects on workflow and clinical decision-making following implementation of the Siemens Healthineers ePOC® Blood Analysis System in a Canadian tertiary-care ED.

## Materials and methods

Objectives

The primary objective of this quality-improvement project was to evaluate clinician-perceived usability, adequacy of training, satisfaction, and perceived effects on workflow and clinical decision-making approximately three months after implementation of the Siemens Healthineers ePOC® Blood Analysis System (ePOC) in a high-volume, tertiary-care academic ED in Canada.

As a secondary objective, we retrospectively extracted core laboratory TATs among encounters in which ePOC testing was also performed, in order to describe the laboratory turnaround interval that bedside testing was intended to bypass. Device-side TATs were not retrievable from the analyzer’s data management system; consequently, a direct ePOC-versus-core-laboratory TAT comparison was not possible, and these data are presented descriptively rather than as a comparative efficiency outcome. This evaluation was not designed as an effectiveness, comparative-efficiency, or diagnostic-accuracy study.

Study design

This was a quality-improvement and implementation evaluation, reported in accordance with the Standards for Quality Improvement Reporting Excellence (SQUIRE) 2.0 guidelines where applicable [10]. It consisted of two components: (1) a retrospective extraction of core laboratory TATs during the early implementation period and (2) a postimplementation survey assessing clinician-reported usability, adequacy of training, satisfaction, and perceived effects on workflow efficiency and clinical decision-making. The two components were combined to situate subjective clinician experience alongside operational data.

Setting

This evaluation was conducted in the ED of St. Michael’s Hospital, a tertiary-care academic hospital in downtown Toronto, Ontario, Canada. The hospital has over 500 inpatient beds, more than 1,500 annual trauma visits, and over 172,000 annual ED visits.

Prior to implementation, blood gas and rapid chemistry testing followed a centralized workflow: a venous or arterial sample was collected in the ED, transported to the core laboratory by porter, prioritized on arrival, analyzed on GEM 4000 analyzers, and reported in the hospital information system. Bedside POCT was introduced with the intent of bypassing the transport and centralized-processing components of this pathway during initial assessment of critically ill and severely injured patients.

Outcomes

TAT Analysis

Study population: Adult patients (≥18 years) presenting to the ED between March and April 2025 for whom blood gas and/or rapid chemistry testing was clinically indicated, as determined by the clinical staff, were eligible for inclusion. Patients were included if POCT using the ePOC device was performed as part of routine clinical care during their ED visit. No additional blood draws were performed for evaluation purposes. This was a convenience sample of consecutive eligible encounters over a fixed time window; no a priori sample size or power calculation was performed.

Implementation and data collection: During a two-week pre-implementation phase, approximately 150 nursing staff and five designated super-users were trained, standard operating procedures were finalized, and devices were configured. Training was delivered in sessions of up to five trainees, with up to one hour of instruction per end user. The super-users provided on-shift troubleshooting and peer support. Five ePOC devices were deployed across the acute and trauma areas of the ED. Bedside POCT and paired-sample collection took place during the study period (March-April 2025), and the device subsequently entered routine ED-wide clinical use in June 2025.

POCT was performed at the bedside using the ePOC device with Siemens ePOC Blood Analysis Test Cards, by trained ED staff, in accordance with institutional standard operating procedures. Quality control was performed weekly, with all three levels of control material run by a medical laboratory assistant or the POCT team. When clinically indicated, a concurrent sample was also sent to the hospital’s core laboratory for standard analysis. TAT data were extracted retrospectively, and core laboratory results were matched to ePOC encounters using EDM Lite by the core laboratory point-of-care team in collaboration with the designated super-users.

Outcome: TAT was defined as the interval from sample collection to result availability in the electronic medical record. Core laboratory TAT was extracted retrospectively and categorized by specimen type (blood gas syringe or plasma) to account for differences in laboratory processing workflows.

Device-side TATs were not retrievable from the ePOC data management system for this evaluation period. A direct within-study comparison of ePOC and core laboratory TAT was therefore not possible, and no pre-implementation baseline of core laboratory TAT was available. TAT data are accordingly reported descriptively, to characterize the laboratory interval during the implementation period.

Postimplementation Clinician Survey

Survey instrument: The survey instrument (the ePOC Clinician Experience Survey) was designed locally by the study team for this evaluation and was not formally validated or piloted. The instrument consisted of 13 items, including clinical role, years in role, perceived importance of individual analytes available on the device, frequency of device use, ease of use, perceived adequacy of training, perceived effects on the efficiency of blood analysis, on time to critical results, and on the speed of clinical decision-making, perceived impact on patient care, overall satisfaction, support for continued ED use, and one optional free-text item inviting additional comments or suggestions. Responses used five-point Likert scales or fixed categorical response options. The full instrument is provided as Supplementary Appendix 1.

Survey participants and administration: Eligible participants included ED staff exposed to the ePOC device during clinical care following implementation, including registered nurses, attending ED physicians, residents, respiratory therapists, anesthesiologists, and trauma team leaders. Participation was voluntary and anonymous, and no incentives were provided.

The ePOC Clinician Experience Survey was administered using SurveyMonkey (Momentive Inc., San Mateo, CA) from August to October 2025, approximately three months after the device entered routine ED-wide use. Recruitment used three parallel channels: (1) an email invitation distributed to eligible ED staff, (2) QR-code posters displayed throughout the ED, and (3) paper copies offered directly to staff by a research assistant during ED shifts.

Data analysis: Quantitative data were analyzed descriptively. Categorical variables were summarized using frequencies and proportions. Denominators varied across items because responses to individual questions were optional; item-specific denominators are reported throughout.

Free-text responses were reviewed and grouped into recurring themes by a single investigator. No formal qualitative coding framework, dual independent coding, or inter-rater reliability assessment was applied, given the small number of free-text responses (12/32) and the descriptive intent of the analysis. Theme counts were not mutually exclusive.

Ethics considerations

This project was conducted as a quality improvement initiative using retrospectively extracted operational data from routine clinical care. No additional patient contact or study-specific testing occurred. The project was approved by the institutional Review of Quality Improvement Studies (ReQuiST) committee on April 25, 2025 (ReQuiST Application #503), which included approval for retrospective extraction of operational data from the preceding implementation period. The ReQuiST committee determined that written informed consent was not required from participants. Survey participation was voluntary and anonymous, and completion of the survey was taken to indicate consent.

Funding and conflicts of interest

Siemens Healthineers provided the ePOC Blood Analysis System devices and test cards used during this implementation period as an in-kind contribution. Siemens Healthineers had no role in the design of this evaluation, the collection or analysis of data, the interpretation of findings, or the decision to submit for publication.

## Results

Between March and April 2025, POCT using the ePOC device was performed in 42 adult patients in the ED. Core laboratory TAT data were available for 28 of these 42 encounters (66.7%) (Table 1). The remaining 14 encounters could not be matched to a core laboratory result, either because the medical record number was entered incorrectly into the device at the point of testing, or because no concurrent core laboratory sample was sent. Among matched encounters, the core laboratory TAT varied by specimen type, with a mean TAT of 18 minutes (range: 0-33) for blood gas syringes and nine minutes (range: 0-19) for plasma samples.

**Table 1:** Core laboratory turnaround time by specimen type among emergency department encounters in which ePOC testing was also performed ePOC: Siemens Healthineers ePOC Blood Analysis System; TAT: turnaround time; min: minutes Values describe core laboratory turnaround time only.

Specimen type	Mean TAT (min)	Range (min)
Blood gas syringe (n = 18)	18	0-33
Plasma sample (n = 10)	9	0-19

Thirty-two ED clinicians completed the postimplementation survey, including 21 nurses (65.6%), 10 physicians (31.3%), and one resident (3.1%) (Table 2). Most respondents reported using the device at least monthly, including 46.9% who reported at least weekly use. Further, most found the tool easy to use, with 65.6% rating it as easy or very easy, 31.2% reporting a neutral experience, and only one respondent (3.1%) reporting difficulty. Perceived adequacy of training was more variable, with 55.2% reporting adequate training and 27.6% inadequate training.

**Table 2:** Clinician respondent characteristics and ePOC device experience ePOC: Siemens Healthineers ePOC Blood Analysis System

Characteristics	n (%)
Clinical role (n = 32)	
Nurse	21 (65.6)
Physician	10 (31.3)
Resident	1 (3.1)
Years in role (n = 32)	
<5 years	13 (40.6)
5-9 years	12 (37.5)
10-14 years	4 (12.5)
15-19 years	1 (3.1)
≥20 years	2 (6.3)
Frequency of ePOC use (n = 32)	
Never	1 (3.1)
Rarely (few times/month)	16 (50.0)
Occasionally (≥1/week)	15 (46.9)
Device ease of use (n = 32)	
Very easy	3 (9.4)
Easy	18 (56.2)
Neutral	10 (31.2)
Difficult	1 (3.1)
Adequacy of training (n = 29)	
Adequate	16 (55.2)
Somewhat adequate	5 (17.2)
Not adequate	8 (27.6)

Respondents rated blood gas parameters, electrolytes, and lactate as highly important in the care of critically ill patients, with 93.8%, 93.8%, and 90.6%, respectively, rating these analytes as very or extremely important (Table 3). Creatinine and hematocrit were less prioritized, with corresponding ratings of 56.3% and 53.1%.

**Table 3:** Clinician-rated importance of laboratory parameters available through ePOC testing (n = 32) ePOC: Siemens Healthineers ePOC Blood Analysis System

Parameter	Extremely important	Very important	Moderately important	Slightly important	Not important
Blood gas	26 (81.3%)	4 (12.5%)	2 (6.3%)	0 (0%)	0 (0%)
Electrolytes	24 (75.0%)	6 (18.8%)	2 (6.3%)	0 (0%)	0 (0%)
Lactate	20 (62.5%)	9 (28.1%)	2 (6.3%)	1 (3.1%)	0 (0%)
Creatinine	12 (37.5%)	6 (18.8%)	8 (25.0%)	5 (15.6%)	1 (3.1%)
Hematocrit	8 (25.0%)	9 (28.1%)	5 (15.6%)	8 (25.0%)	2 (6.3%)

Respondents generally reported a positive impact on workflow (Table 4). Reported improvements were common for efficiency of blood analysis (80.0%), time to critical results (80.6%), and speed of clinical decision-making (83.3%). Overall satisfaction was also high, with 75.0% reporting that they were satisfied or very satisfied with the device. Most respondents felt that the device had improved patient care, with 22 of 32 (68.8%) answering yes, compared to three (9.4%) no and seven (21.9%) unsure. Satisfaction with the device was also favorable, as 18 respondents (56.3%) reported being satisfied and six (18.8%) very satisfied, while eight (25.0%) were neutral and none reported dissatisfaction. Support for continued use in the ED was particularly strong, with 29 of 32 respondents (90.6%) indicating that the ePOC device should continue to be used, compared with one (3.1%) no and two (6.3%) unsure.

**Table 4:** Clinician-reported impact of ePOC testing on ED workflow metrics ePOC: Siemens Healthineers ePOC Blood Analysis System; ED: emergency department Responses to five-point items were collapsed into improved, unchanged, and worsened

ED workflow metric	Improved (%)	Unchanged (%)	Worsened (%)
Efficiency of blood analysis (n = 30)	24 (80.0)	5 (16.7)	1 (3.3)
Time to critical results (n = 31)	25 (80.6)	3 (9.7)	3 (9.7)
Speed of clinical decision-making (n = 30)	25 (83.3)	3 (10.0)	2 (6.7)

Optional free-text comments were provided by 12/32 respondents (37.5%). The most common themes were limited training or device access (n = 4), delays related to analyzer calibration (n = 3), limitations in result transfer or documentation (n = 3), and concerns about agreement between ePOC device and core laboratory values (n = 3). Positive comments were also noted (n = 3), including ease of use, rapid access to electrolyte results, and utility in time-sensitive clinical situations. Theme counts were not mutually exclusive.

## Discussion

In this early implementation evaluation of the ePOC analyzer in a tertiary-care Canadian ED, clinicians reported generally positive views of the device, particularly regarding its perceived effects on workflow efficiency, time to critical results, and speed of clinical decision-making. Overall satisfaction was high, and most respondents supported continued use in the ED. However, reported frequency of use varied, and several barriers to routine use were identified, including perceived inadequacy of training, calibration delays, difficulty transferring results into the electronic medical record, and concerns about agreement with core laboratory values.

Survey respondents placed greater importance on blood gas parameters, electrolytes, and lactate than on creatinine and hematocrit, suggesting that clinicians may have viewed the device primarily as a tool for early physiologic assessment rather than for broader biochemical workup. The ePOC underwent weekly quality control with control materials, but a local method comparison against the core laboratory analyzers was not performed as part of this evaluation. Published method-comparison data from a Toronto center have reported mean bias below 5% for blood gases, electrolytes, and lactate, with larger and more variable differences for creatinine and hematocrit [11]. This pattern aligns with the lower importance our respondents placed on creatinine and hematocrit and may explain the agreement concerns raised later in free-text comments.

Respondents also perceived improvements in the efficiency of blood analysis, time to critical results, and speed of clinical decision-making. These perceptions are consistent with previous work reporting that POCT improves timeliness when testing is performed early in assessment [2-5]. Since we only measured clinician perceptions, we cannot confirm if a time advantage was realized in our setting.

The barriers identified here are consistent with those described in the broader POCT implementation literature, in which device connectivity, documentation workflows, and competency maintenance are recurring obstacles to sustained uptake [12,13]. More than one-quarter of respondents perceived training as inadequate despite a structured pre-implementation training programme with designated super-users, which suggests that initial training alone may be insufficient to sustain competence across a large, rotating ED workforce. Randomized and pathway-based studies have reported improvements in laboratory TAT, time to clinical decision, and ED length of stay when POCT is embedded within standardized care processes rather than deployed as a stand-alone adjunct [3,14,15]. In our setting, this includes local evidence-based guidelines for use of the ePOC device and addition of blood gas POCT into standardized care [16]. Additionally, repeated training sessions, ongoing competency support, and clear documentation processes can sustain uptake of the ePOC device [17,18].

This evaluation has several important limitations. First, it was conducted at a single centre, and findings may not generalize to EDs with different patient volumes, staffing models, or laboratory turnaround performance. Second, the TAT component does not constitute a comparative efficiency analysis. Device-side TATs were not retrievable from the analyzer’s data management system, and no pre-implementation baseline of core laboratory TAT was available. The reported values therefore describe core laboratory performance during the implementation period only. Third, the two data components were not concurrent. TAT data were collected during the early implementation and training period (March-April 2025), whereas the survey was administered from August to October 2025, following ED-wide rollout in June 2025. The two datasets therefore capture different points on the adoption curve and should not be interpreted as describing a single implementation state. Fourth, 14 of 42 encounters (33.3%) could not be matched to a core laboratory result. We could not verify that these unmatched encounters were missing at random, and if they differed systematically from the matched encounters, the matched subset would not be representative of all encounters in which the device was used. Fifth, because the survey was anonymous and distributed through email, QR code posters, and paper copies, the number of clinicians invited could not be established, and a response rate could not be calculated. The potential for nonresponse bias therefore cannot be quantified, and respondents may have been those with the strongest views about the device in either direction. In addition, survey items had varied denominators because individual items were optional, and the reasons for item nonresponse were not captured. Nearly half of respondents were infrequent users, which may have influenced reported perceptions of utility and barriers, and some items may have been interpreted differently depending on clinical role and familiarity with the device. Sixth, free-text responses were provided by a minority of respondents (12/32) and were grouped into themes by a single investigator without a formal qualitative coding framework or assessment of inter-rater reliability.

## Conclusions

In this tertiary-care ED, early implementation of a point-of-care blood gas and chemistry analyzer was met with favorable clinician perceptions of efficiency, usability, and support for clinical decision-making. However, variable uptake and reported barriers, including inadequate training, calibration delays, and limited electronic medical record integration, suggest that the potential benefits of bedside testing may not be consistently realized in routine care without structured implementation support. Clear indications for use, integration into standardized ED workflows, ongoing training and competency assessment, and local analytical verification are likely to be important, although not assessed here. Future evaluations should examine how structured implementation strategies influence the clinical uptake of point-of-care blood testing as well as downstream measures of emergency care delivery and patient outcomes.

## Appendices

Supplementary appendix 1: ePOC clinical staff experience survey

1.     What is your primary clinical role?

a.     Nurse

b.    Physician

c.      Respiratory therapist

d.     Other (describe)

2.     How many years have you been in your role?

a.     <5 years

b.     5-9 years

c.      10-14 years

d.     15-19 years

e.     20 or more years

3.     How important are the following labs for a critically unwell or severely injured patient?

a.     List the lab values on ePOC with associated Likert scale 1-5 (not important to extremely important)

**Table 5:** Item 3-importance of laboratory parameters

Parameter	Not important	Slightly important	Moderately important	Very important	Extremely important
Blood gas	☐	☐	☐	☐	☐
Electrolytes	☐	☐	☐	☐	☐
Lactate	☐	☐	☐	☐	☐
Creatinine	☐	☐	☐	☐	☐
Hematocrit	☐	☐	☐	☐	☐

4.     On average over the last month, how often have you been involved in the care of a patient where the ePOC device was used?

a.     Frequently (i.e., once or more per shift)

b.     Occasionally (i.e., once or more per week)

c.      Rarely (i.e., a few times over the month)

d.     Never

5.     How would you rate the ease of use of the device?

a.     Very difficult

b.     Difficult

c.      Neutral

d.     Easy

e.     Very easy

6.     Do you feel like you were adequately trained on the device?

a.     Yes

b.     No

c.      Somewhat

7.     How has the device impacted the efficiency of the blood analysis process? (Likert scale: greatly decreased efficiency to greatly increased efficiency)

**Table 6:** Item 7-perceived impact on efficiency of blood analysis

Greatly decreased efficiency	Somewhat decreased efficiency	No change	Somewhat increased efficiency	Greatly increased efficiency
☐	☐	☐	☐	☐

8.     How has ePOC testing impacted the time to obtaining CRITICAL lab results for patients? (Likert scale significantly increased to significant reduced)

**Table 7:** Item 8-perceived impact on time to critical results

Significantly increased time	Somewhat increased time	No change	Somewhat reduced time	Significantly reduced time
☐	☐	☐	☐	☐

9.     How has ePOC testing impacted the speed of clinical decision making? (Likert scale greatly slowed to greatly improved decision making)

**Table 8:** Item 9-perceived impact on speed of clinical decision-making

Greatly slowed decision-making	Somewhat slowed decision-making	No change	Somewhat improved decision-making	Greatly improved decision-making
☐	☐	☐	☐	☐

10.  Do you feel like the device has improved patient care?

a.     Yes

b.     No

c.      Unsure

11.  Overall, how satisfied are you with ePOC? (Likert scale: very dissatisfied to very satisfied)

**Table 9:** Item 11-overall satisfaction with ePOC

Very dissatisfied	Somewhat dissatisfied	Neither satisfied nor dissatisfied	Somewhat satisfied	Very satisfied
☐	☐	☐	☐	☐

12.  Should we continue to use ePOC in the emergency department?

a.     Yes

b.     No

c.      Unsure

13.  Please provide any additional comments or suggestions regarding the device (optional):
